# Heavy Metal Stress and Some Mechanisms of Plant Defense Response

**DOI:** 10.1155/2015/756120

**Published:** 2015-01-26

**Authors:** Abolghassem Emamverdian, Yulong Ding, Farzad Mokhberdoran, Yinfeng Xie

**Affiliations:** ^1^Center of Modern Forestry in Southern China, Nanjing Forestry University, Nanjing 210037, China; ^2^College of Biology and the Environment, Nanjing Forestry University, Nanjing 210037, China; ^3^Bamboo Research Institute, Nanjing Forestry University, Nanjing 210037, China; ^4^Department of Agronomy and Plant Breeding, Faculty of Agriculture, Islamic Azad University, Mashhad Branch, Mashhad 9187147578, Iran

## Abstract

Unprecedented bioaccumulation and biomagnification of heavy metals (HMs) in the environment have become a dilemma for all living organisms including plants. HMs at toxic levels have the capability to interact with several vital cellular biomolecules such as nuclear proteins and DNA, leading to excessive augmentation of reactive oxygen species (ROS). This would inflict serious morphological, metabolic, and physiological anomalies in plants ranging from chlorosis of shoot to lipid peroxidation and protein degradation. In response, plants are equipped with a repertoire of mechanisms to counteract heavy metal (HM) toxicity. The key elements of these are chelating metals by forming phytochelatins (PCs) or metallothioneins (MTs) metal complex at the intra- and intercellular level, which is followed by the removal of HM ions from sensitive sites or vacuolar sequestration of ligand-metal complex. Nonenzymatically synthesized compounds such as proline (Pro) are able to strengthen metal-detoxification capacity of intracellular antioxidant enzymes. Another important additive component of plant defense system is symbiotic association with arbuscular mycorrhizal (AM) fungi. AM can effectively immobilize HMs and reduce their uptake by host plants via binding metal ions to hyphal cell wall and excreting several extracellular biomolecules. Additionally, AM fungi can enhance activities of antioxidant defense machinery of plants.

## 1. Introduction

Anthropogenic perturbations of biosphere manifested in a broad array of global phenomena including accelerated rate of industrialization, intensive agriculture, and extensive mining accompanied by burgeoning population and rapid urbanization have not only wreaked the havoc on the availability of natural resources but also caused widespread and grave contamination of essential components of life on the planet. Of the implications of human-induced disturbance of natural biogeochemical cycles, accentuated accumulation of heavy metals (HMs) is a problem of paramount importance for ecological, nutritional, and environmental reasons [1, 2]. HMs belong to group of nonbiodegradable, persistent inorganic chemical constituents with the atomic mass over 20 and the density higher than 5 g·cm^−3^ that have cytotoxic, genotoxic, and mutagenic effects on humans or animals and plants through influencing and tainting food chains, soil, irrigation or potable water, aquifers, and surrounding atmosphere [3–6]. There are two kinds of metals found in soils, which are referred to as essential micronutrients for normal plant growth (Fe, Mn, Zn, Cu, Mg, Mo, and Ni) and nonessential elements with unknown biological and physiological function (Cd, Sb, Cr, Pb, As, Co, Ag, Se, and Hg) [5, 7–9]. Both underground and aboveground surfaces of plants are able to receive HMs [10]. The essential elements play a pivotal role in the structure of enzymes and proteins. Plants require them in tiny quantities for their growth, metabolism, and development; however, the concentration of both essential and nonessential metals is one single important factor in the growing process of plants so that their presence in excess can lead to the reduction and inhibition of growth in plants [11]. HMs at toxic levels hamper normal plant functioning and act as an impediment to metabolic processes in a variety of ways, including disturbance or displacement of building blocks of protein structure, which arises from the formation of bonds between HMs and sulfhydryl groups [12], hindering functional groups of important cellular molecules [13], superseding or disrupting functionality of essential metals in biomolecules such as pigments or enzymes [2], and adversely affecting the integrity of the cytoplasmic membrane [14], resulting in the repression of vital events in plants such as photosynthesis, respiration, and enzymatic activities [13]. On the other hand, elevated levels of HMs are associated with the increased generation of reactive oxygen species (ROS), such as superoxide free radicals (O_2_^•−^), hydroxyl free radicals (OH^•−^), or non-free radical species (molecular forms) such as singlet oxygen (O_2_^*^) and hydrogen peroxide (H_2_O_2_) as well as cytotoxic compounds like methylglyoxal (MG), which can cause oxidative stress via disturbing the equilibrium between prooxidant and antioxidant homeostasis within the plant cells [11, 13, 15]. This condition implicates the causation of multiple deteriorative disorders such as, oxidation of protein and lipids, ion leakage, oxidative DNA attack, redox imbalance, and denature of cell structure and membrane, ultimately resulting in the activation of programmed cell death (PCD) pathways [1, 3, 5, 16–18]. Plants employ various inherent and extrinsic defense strategies for tolerance or detoxification whenever confronted with the stressful condition caused by the high concentrations of HMs. As a first step towards dealing with metal intoxication, plants adopt avoidance strategy to preclude the onset of stress via restricting metal uptake from soil or excluding it, preventing metal entry into plant root [19]. This can be achieved by some mechanisms such as immobilization of metals by mycorrhizal association, metal sequestration, or complexation by exuding organic compounds from root [10, 20]. At next stage, if these strategies fail and HMs manage to enter inside plant tissues, tolerance mechanisms for detoxification are activated which include metal sequestration and compartmentalization in various intracellular compartments (e.g., vacuole) [10], metal ions trafficking, metal binding to cell wall, biosynthesis or accumulation of osmolytes andosmoprotectants, for example, proline (Pro), intracellular complexation or chelation of metal ions by releasing several substances, for example, organic acids, polysaccharides, phytochelatins (PCs), and metallothioneins (MTs) [20–23], and eventually if all these measures prove futile and plants become overwhelmed with toxicity of heavy metal (HM), activation of antioxidant defense mechanisms is pursued [24]. This review has attempted a comprehensive account of past developments and current trends using more than 235 articles in the research on HM poisoning in plants, exploring the response of vital growth, morphological, anatomical, production, and physiological parameters of plants to HM toxicity as well as investigating detoxifying roles of some defense mechanisms adopted by plants in the face of trace element excess. The study also focuses on underlying functions and detoxification capabilities of two important ligand peptides including PCs and MTs that are typically used by plants to enhance tolerance to HM toxicity. Moreover, another line of plant defense strategy to combat against toxicity of HMs which involves the utilization of primary metabolite molecule Pro and the instigation of AM symbiotic system as well as their possible collaboration with one another or with plant antioxidant system is discussed and surveyed. Lastly, some suggestions will be made in terms of pursuing ways to have a better understanding of metal-plant causality involving action and reaction between these two abiotic and biotic entities in ever-changing natural environments and climate. Some directions for future works will also be provided.

## 2. Effects of Some HMs on Plants

Bioactive-metals, based on their physicochemical properties, are divided into two groups of redox-active metals such as Cr, Cu, Mn, and Fe and non-redox active metals such as Cd, Ni, Hg, Zn, and Al [25, 26]. The redox metals can directly generate oxidative injury via undergoing Haber-Weiss and Fenton reactions, which leads to the aforementioned production of ROS or oxygen free radicals species in plants, resulting in cell homeostasis disruption, DNA strand breakage, defragmentation of proteins, or cell membrane and damage to photosynthetic pigments, which may trigger cell death [7, 27]. In contrast, non-redox active metals indirectly inflict oxidative stress via multiple mechanisms including glutathione depletion, binding to sulfhydryl groups of proteins [25], inhibiting antioxidative enzymes, or inducing ROS-producing enzymes like NADPH oxidases [28]. The basic criterion for the selection of HMs for this review study was based on their mode of action in biological system of plants, whether they are redox active or inactive metals in plant cells. Therefore, three metals (Cu, Cr, and Mn) that are known for taking part in redox reactions in plants and three non-redox active metals (Ni, Zn, and Al) are reviewed in detail to show how they impinge on plants despite possessing different redox states.

### 2.1. Chromium (Cr)

It is well documented that Cr is a toxic agent for the growth and development of plants [29–31]. In addition, it is known as one of the causes of environmental pollution [32]. In plants, Cr is found in the forms of trivalent Cr^3+^ and hexavalent Cr^6+^ species, where the former is of lower toxicity than the latter. Cr is transported and accumulated via carrier ions such as sulfate or iron and is not directly absorbed by plants [32, 33]. Moreover, under reducing condition, Cr^6+^ is converted to its more toxic form Cr^3+^, which can indirectly influence and change soil pH to both alkalinity or acidity extremes, depending on prevailing condition in soil subsurface [34]. This phenomenon might perturb the nutrients bioavailability and their sorption by plants. The highest concentration of Cr occurs in the root rather than other parts of plants [35].

Immobilization of chromium in vacuole of plant root cells is suggested as a main reason for the excessive accumulation of this metal in roots [36, 37]. Cr drastically reduces seedling dry matter production and hampers the development of stems and leaves during plant early growth stage [37]. Chromium toxicity inhibits the cell division and elongation of plant roots, thus shortening the overall length of roots [38]. As a consequence, water and nutrient absorption processes are severely restricted, which can lead to the decreased shoot growth. Furthermore, the extended cell cycle is attributed to toxic presence of Cr in roots [39]. Fozia et al. [40] assessing effects of chromium on growth attributes of sunflower (*Helianthus annuus* L.) ascribed the observed decrease in root length to cell cycle extension triggered by Cr toxicity. Cr can cause damage to plants through manipulating some mechanisms that occur inside cell. Zou et al. [30] in an in vitro study observed that Cr reduced root growth of* Amaranthus viridis* L. This decrease was associated with cell division inhibition and imbalance of Ca^2+^ in cells caused by Cr, disturbing the transport of calcium cation from plasma membrane into cytoplasm. Chromium is capable of creating metabolic disorders during seed germination by disrupting the events related to the conversion of food reservoirs into energy that are essential for the subsequent successful emergence and establishment of seedlings. In cowpea (*Vigna sinensis* (L.) savi ex Hassk) seeds, treated with different concentrations of Cr^6+^, amylase activity and total amount of sugar were markedly decreased, resulting in depression of germination characteristics [41]. Nagajyoti et al. [1] also reported that increased levels of Cr in different valence states were associated with concomitant decline in seed germination. This can be due to disruption in carbohydrate transfer into embryonic axis in seeds or a rise in protease activity [42]. Both antagonistic and synergistic interactions between chromium levels and content of different elements in parts of plants are observed. Samantaray et al. [43] reported that chromium is involved in interfering with the absorption or accumulation of a wide range of other metals or nutrients such as Fe, Mn, Ca, Mg, K, and P in both aerial or root parts of plants, mostly leading to their reduced cellular or tissue concentration. Živković et al. [44] carried out a study on the effects of different trace metals on three* Veronica* species (Plantaginaceae) and found a high positive correlation between Fe and Cr concentration in plant tissues.

### 2.2. Aluminum (Al)

Al is known as an inhibitory element for the growth of plants, especially in acidic soils with pH values as low as 5 or 5.5 where the most phytotoxic form of aluminum (Al^3+^) is prevalent [45]. Although there is still no known or proven biological role for aluminum in plants, some reports demonstrate that Al at low concentrations may lead to the stimulation of plant growth [46]. A 2-3 *μ*g·g^−1^ aluminum threshold in soils with a pH below 5.5 is considered to be hazardous to most plants [47]. The primary target of Al toxicity is roots of plants where the accumulation of Al inflicts the inhibition of root growth in the space of minutes or hours [48]. It can increase the thickness of lateral roots and change their color to brown [49]. Reduction of root respiration and disturbances in the enzymatic regulation of sugar phosphorylation are also caused by Al toxicity [50]. The molecular events responsible for Al-induced root depression may include the attachment of Al to carboxylic groups of pectins in root cells [51], or the hindrance of cell division in roots by Al ions binding to DNA, which results in the enhanced structural rigidity of double helix in DNA and cell wall [52]. Al toxicity stress negatively affects aerial parts of plants, especially as a result of initial root damage [53], which hampers nutrient uptake ability of roots, resulting in nutritional deficiency [54] but the symptoms are not as conspicuous as those observed in roots [55].

The symptomatic effects of Al-induced stress on shoots, which are similar to phosphorus deficiency, may be stunting of leaves, purple discoloration on stems, leaves, and leaf veins followed by yellowing and dead leaf tips [56], and those that resemble calcium deficiency can be curling or rolling of young leaves and death of growing points or petioles [55]. The other visible indications of Al toxicity are the appearance of small necrotic spots on the border of young leaves and chlorosis in the margins and center of older leaves [53]. The reduction in stomatal aperture and decreased photosynthetic activity are also reported to be caused by Al toxicity [57]. Bhalerao and Prabhu [58] reported that Al toxicity in plants such as maize (*Zea mays* L.) and sorghum (*Sorghum bicolor* (L.) Moench) can lead to perturbation in the absorption and transportation of some major nutrients including P, K, Ca, and Mg. A range of morphological and physiological responses have been observed in crop plants when they are exposed to different levels of aluminum. Hossain et al. [59] studying two wheat (*Triticum aestivum* L.) cultivars varying in their degree of sensitivity to Al stress found that the length of root in Al-sensitive cultivar was conspicuously decreased. Moreover, Al stress especially in sensitive cultivar raised the amount of some cellular substances such as pectin and hemicelluloses. Batista et al. [60] observed that leaf sheaths of corn plants treated with different doses of Al had underdeveloped epidermal and cortical cells, which was accompanied by a decrease in the diameter of metaxylem and protoxylem in vascular bundle. At the ultrastructural level, alteration of chromatin configuration in nucleus and an increase in the size and frequency of nucleolar vacuoles are ascribed to Al stress [61].

### 2.3. Manganese (Mn)

Mn is an essential micronutrient that plays a pivotal part in many metabolic and growth processes in plants including photosynthesis, respiration, and the biosynthesis of enzymes such as malic enzyme, isocitrate dehydrogenase, and nitrate reductase [62]. It is also a cofactor required for multiple plant enzymes, for example, Mn-dependent superoxide dismutase (MnSOD) [63]. Furthermore, manganese is involved in carbohydrate and nitrogen metabolism, synthesis of fatty acid, acyl lipids, and carotenoid as well as hormonal activation [33, 63, 64]. The contribution of manganese to the functionality of photosystem II (PSII) especially during the course of splitting of water molecules into oxygen [65] and its role in the protection of PSII from photo damage are of significant importance [66]. Mn^2+^ is the most stable and soluble form of manganese in the soil environment [67]. However, lower soil pH, less soil organic matter, and decreased redox potential increase the availability or toxicity of Mn^2+^ to plants [67, 68]. Contrary to some elements such as aluminum or copper, there is a tendency for manganese to easily translocate form roots to the upper parts of plants. This mobility is the reason why symptoms of Mn toxicity are first visible in aerial organs of plants [69]. The appearance of visual features in plants affected by Mn toxicity varies with the type of plant species, plant age, temperature, and light level [70–72]. The symptoms may include crinkled leaves [73], darkening of leaf veins on older foliage [74], chlorosis and brown spots on aged leaves [75], and black specks on the stems [76]. Mn toxicity has been associated with a decreased CO_2_ assimilation but unaffected chlorophyll (Chl) level in* Citrus grandis* seedlings [77] and depleted Chl content in pea (*Pisum sativum* L.) [78] and soybean (*Glycine max* L.) [79], indicating diversity among plant species in response to Mn excess.

Combined effects of excessive manganese and light on plants have received particular attention in the literature. González et al. [70] examining the interactive effects of light intensity and Mn excess on two Mn-susceptible and tolerant genotypes of common bean (*Phaseolus vulgaris* L.) demonstrated that high light aggravated the toxic influence of Mn, causing the plants to produce, respectively, a 270% and 130% more ascorbate peroxidase (APX) in leaves to cope with Mn toxicity. In maple trees (*Acer platanoides*) leaves exposed to intense sun light had more concentrations of Mn than shade leaves [80]. In contrast, Hajiboland and Hasani [81] working with rice (*Oryza sativa* L.) and sunflower found that although Mn toxicity depressed shoot and root growth, the intensification of light diluted concentration of Mn in these plants, ameliorating growth inhibition caused by Mn treatment. Wissemeier and Horst [82] in cowpea (*Vigna unguiculata* Walp) found that light intensity did not play any part in exacerbating adverse effects of Mn toxicity and in fact it was low light that accelerated the expression of toxic symptoms of Mn. It seems that the negative impacts of Mn toxicity are alleviated or accentuated by different light levels, depending on plant variation and tolerance.

### 2.4. Nickel (Ni)

Ni is a micronutrient that is required by both higher and lower plants in very small amounts [83] but its phytotoxicity is deemed to be more important than its shortage [84]. Ni has various oxidative states but its divalent state (Ni^2+^) is the most stable type in the environment and biological systems [85]. Although the role of Ni in metabolic processes of plants has not been identified as extensively as other elements such as Mn or Cu, it is a key factor in the activation of enzyme urease, which is needed for nitrogen metabolism [86]. Moreover, it plays a part in seed germination and iron uptake [85]. The concentration level representing nickel toxicity in plants varies greatly from 25 to 246 *μ*g·g^−1^ dry weight (DW) of plant tissue, depending on the plant species and cultivars [87]. Ni at excess competes with several cations, in particular, Fe^2+^ and Zn^2+^, preventing them from being absorbed by plants, which ultimately causes deficiency of Fe or Zn and results in chlorosis expression in plants [88].

Excess nickel adversely affects germination process and seedling growth traits of plants by hampering the activity of the enzymes such as amylase and protease as well as disrupting the hydrolyzation of food storage in germinating seeds [89, 90]. Plant growth parameters and attributes are also affected by Ni toxicity. M. R. Khan and M. M. Khan [88] investigating the toxic effect of nickel and cobalt on chickpea (*Cicer arietinum* L.) showed that toxicity of Ni on the biomass production was more pronounced than Co and both metals led to poor nodulation, resulting in the reduced yield. Al-Qurainy [91] also demonstrated that Ni at the concentration 150 *μ*g·g^−1^ of soil severely reduced plant height and leaf area in bean.

Ni, especially at high concentrations, can readily move through phloem and xylem vessels, thereby translocating smoothly from the root to the upper parts of plants [92]. This ease of movement towards shoots is due to the pattern by which Ni is distributed within the tissue of roots, which differs from some other HMs such as Pb and Cd so that it can pass through the endodermal barrier and amass in the pericycle cells [93]. Several studies in plants including maize [94] and cowpea [95] have confirmed this phenomenon and indicated that Ni toxicity can result in inhibited lateral root formation and development. Moreover, the agglomeration of Ni in root apex greatly hampers mitotic cell division in this organ, which ultimately results in growth reduction [96]. The induction of ROS, due to Ni toxicity, is observed in both agronomic and nonagronomic plants such as* Jatropha curcas* L. [97], or wheat [98], which results in a wide range of physiological and biological disorders including the impairment of cell membrane and enzymatic imbalance.

The adverse impact of toxic levels of Ni on the photosynthetic apparatus and performance is conspicuous. Sreekanth et al. [99] reported that Ni toxicity can lead to reduced Chl content and interruption of electron transport. Ghasemi et al. [100] in maize (*Zea mays* L.) showed that excess Ni perniciously influenced photosynthetic protein complexes and the rate of Hill reaction dwindled by increasing Ni concentration.

### 2.5. Copper (Cu)

Cu is an essential micronutrient that participates in many vital physiological functions of plants including acting as a catalyzer of redox reaction in mitochondria, chloroplasts, and cytoplasm of cells [101] or as an electron carrier during plant respiration [102]. However, Cu becomes toxic when its concentration in the tissue of plants rises above optimal levels [103]. Cu exists in many states in soils but is mainly taken up by plants in the form of Cu^2+^ [104]. The concentration of copper in soil is typically between 2 and 250 *μ*g·g^−1^ and healthy plants can absorb 20–30 *μ*g·g^−1^ DW [105]. But copper availability depends greatly on soil pH and its phytoavailability increases with declining pH [106]. In addition, uptake of Cu by plants and its toxicity are contingent on nutritional condition of plant, Cu^2+^ concentration in soil, length of exposure, and genotype of a species [107]. A plethora of research studies such as [106] in Rhodes grass (*Chloris gayana* Knuth), [108] in clove (*Syzygium aromaticum* L.), [109] in cucumber (*Cucumis sativus*), and [110] in some* Eucalyptus* species indicate that copper has a propensity for the accumulation in the root tissues with little upward movement towards shoots. Therefore, the initial characterization of Cu toxicity is the hindrance of root elongation and growth [111]. The subsequent symptoms include chlorosis, necrosis, and leaf discoloration [102]. Excess Cu can become attached to the sulfhydryl groups of cell membrane or induce lipid peroxidation, which results in the damaged membrane and the production of free radicals in different plant organelles and parts [112]. Of these pernicious effects, damage to the permeability of root cells [113] and structural disturbance of thylakoid membranes [114] can be mentioned. Cu at toxic levels through redox process cycling between Cu^+^ and Cu^2+^ triggers the formation of reactive oxygen species such as singlet oxygen (O_2_^−^) and hydroxyl radical (HO^•^), leading to injuries to macromolecules, for example, DNA, RNA, lipids, carbohydrates, and proteins [103, 115]. Decreased photosynthetic competence, low quantum efficiency of PSII, and reduced cell elongation are also associated with Cu toxicity [109]. These trends have been observed in various levels of copper applied to different plants. In an in vivo study of bean (*Phaseolus vulgaris* L. cv. Dufrix), it was shown that toxic concentration of Cu (15 *μ*M) depleted PSII action centers and led to photoinhibition and disruption of its repair cycle [116]. Moreover, the results obtained with rapeseed (*Brassica napus* L.) indicated that content of chlorophylls (Chl *a* and Chl *b*) as well as carotenoids was markedly dropped when this plant was exposed to 6 *μ*mol·dm^−3^ concentration of Cu [117]. Seedling growth characteristics are shown to be adversely affected by Cu toxicity. Sharma et al. [118] working with spinach seeds (*Spinacia oleracea* L.) found a significant negative correlation between the root and shoot elongation with increasing Cu levels, which was associated with a noticeable depression in seedling fresh weight. Mediouni et al. [119] comparing effects of cadmium and copper toxicity on tomato seedling (*Lycopersicon esculentum* Ibiza F1) observed that Cu and Cd significantly decreased biomass production of tomato. Also, Cu toxicity was found to be more pronounced and resulted in more induction of lipid peroxidation in the young seedlings, especially at high concentrations, than that of Cd.

### 2.6. Zinc (Zn)

Zn is an essential trace metal that despite having no redox activity is particularly involved in many vital physiological events in plants [120]. Zinc is an indispensable component of special proteins known as zinc fingers that bind to DNA and RNA and contribute to their regulation and stabilization [121]. Moreover, it is a constituent of various enzymes, for example, oxidoreductases, transferases, and hydrolases [114], as well as ribosome [122], and plays a role in the formation of carbohydrates and chlorophyll and root growth [123].

Zinc, in divalent state (Zn^2+^), is the most pervasive form found in soil and acquired by plants [124]. Zn bioavailability/phytoavailability is dependent on various variables including the total Zn concentration in soil, lime content and organic matter of soil, clay type, and presence of other HMs, soil's pH, and the amount of salt in the substrate [125, 126]. Of these, pH is the most important factor influencing Zn availability [124] and higher pH is generally associated with the decreased absorption of Zn by plants [126]. Zn at high soil concentrations (150 to 300 *μ*g·g^−1^) is strongly toxic [127] and its phototoxicity, in addition to the bioavailability factors, depends on plant type and plant development stage [128]. Visual signs of trouble in plants as a result of Zn toxicity are reported to be chlorosis in young leaves due to iron or manganese deficiency [129] and appearance of purplish-red color in leaves due to phosphorus deficiency [127], which indicate that Zn^2+^ in excess can easily supersede other metals, especially those with similar ionic radii in the active sites of enzymes or transporters [130]. Moreover, necrotic spotting between the veins in the blade of mature leaves [131] and inward rolling at leaf margins [120] are attributed to Zn toxicity.

Excess Zn^2+^ in cells can produce ROS and adversely influence integration and permeability of membrane [132, 133]. Zn toxicity, akin to other HMs, hampers the functionality and efficiency of photosynthetic system in different plant species. Vassilev et al. [134] in bean plants, Mirshekali et al. [135] in sorghum (*Sorghum bicolor* L.), and lalelou et al. [136] in naked pumpkin (*Cucurbita pepo*) showed that excessive concentration of Zn^2+^ reduced the content of accessory photosynthetic pigments including Chl *a* and Chl *b* by disturbing the absorption and translocation of Fe and Mg into chloroplast. The elevated level of Zn^2+^ is reported to cause a decline in initial and maximum Chl fluorescence, resulting in the repression of PSII activity [137]. Zinc in excess is found to have genotoxic effects on plants, resulting in genetic-related disorders and damages to plants. Oladele et al. [138] demonstrated that high levels of Zn (100 mg·L^−1^) in cells resulted in abnormal chromosomes, which was followed by a sticky metaphase and premature separation of chromosomes in bambara groundnut (*Vigna subterranean*). Also, Truta et al. [139] observed that the rate of ana-telophase aberrations was 2-3 times higher than control treatment when barely seedlings (*Hordeum vulgare* L.) were treated with 250 to 500 *μ*M Zn^2+^.

Growth parameters and structure of plant parts are shown to be negatively affected by Zn toxicity. Todeschini et al. [140] demonstrated that Zn in poplar (*Populus alba*) drastically changed leaf morphology and ultrastructure and caused the formation of calcium-oxalate crystals. Vijayarengan and Mahalakshmi [141] showed that Zn toxicity decreased the length of root and shoot as well as area of leaves in tomato (*Solanum lycopersicum* L.).

## 3. Some Defense Mechanisms Employed by Plants against HM Stress

As mentioned earlier, plants possess a sophisticated and interrelated network of defense strategies to avoid or tolerate HM intoxication. Physical barriers are the first line of defense in plants against metals. Some morphological structures like thick cuticle, biologically active tissues like trichomes, and cell walls as well as mycorrhizal symbiosis can act as barriers when plants are faced with HM stress [12, 142, 143]. Trichomes, for instance, can either serve as HM storage site for detoxification purposes or secrete various secondary metabolites to negate hazardous effects of metals [144, 145]. On the other hand, once HMs overcome biophysical barriers and metal ions enter tissues and cells, plants initiate several cellular defense mechanisms to nullify and attenuate the adverse effects of HMs. Biosynthesis of diverse cellular biomolecules is the primary way to tolerate or neutralize metal toxicity. This includes the induction of a myriad of low-molecular weight protein metallochaperones or chelators such as nicotianamine, putrescine, spermine, mugineic acids, organic acids, glutathione, phytochelatins, and metallothioneins or cellular exudates such as flavonoid and phenolic compounds, protons, heat shock proteins, and specific amino acids, such as proline and histidine, and hormones such as salicylic acid, jasmonic acid, and ethylene [19, 20, 146]. When the above-mentioned strategies are not able to restrain metal poisoning, equilibrium of cellular redox systems in plants is upset, leading to the increased induction of ROS [147]. To mitigate the harmful effects of free radicals, plant cells have developed antioxidant defense mechanism which is composed of enzymatic antioxidants like superoxide dismutase (SOD), catalase, (CAT), ascorbate peroxidase (APX), guaiacol peroxidase (GPX), and glutathione reductase (GR) and nonenzymatic antioxidants like ascorbate (AsA), glutathione (GSH), carotenoids, alkaloids, tocopherols, proline, and phenolic compounds (flavonoids, tannins, and lignin) that act as the scavengers of free radicals [18, 148, 149]. As previously indicated, some of the biological molecules involved in cellular metal detoxification can be multifunctional and have antiradical, chelating, or antioxidant activities. Exploitation and upregulation of any of these mechanisms and biomolecules may depend on plant species, the level of their metal tolerance [150], plant growth stage, and metal type. Some of the defense mechanisms used by plants against HMs will be discussed below.

### 3.1. Phytochelatins (PCs)

One of the mechanisms adopted by plants to detoxify HMs is the production of short-chain thiol-rich repetitions of peptides of low-molecular weight synthesized from sulfur-rich glutathione (GSH) by the enzyme phytochelatin synthase (PCS) with the general structure of (*γ*-glutamyl-cysteinyl) *n*-glycine (*n* = 2 to 11) that have a high affinity to bind to HMs when they are at toxic levels [151–155]. Phytochelatins, as a pathway for metal homeostasis and detoxification, have been identified in a wide range of living organisms from yeast and fungi to many different species of animals [156, 157]. In plants, PCs are found to be part of the defensive act not only against metal-related stresses but also in response to other stressors such as excess heat, salt, UV-B, and herbicide [158]. PCs are reported to be used as biomarkers for the early detection of HM stress in plants [159]. Cytosol is the place where PCs are manufactured and actively shipped from there in the form of metal-phytochelatin complexes of high molecular weight to vacuole as their final destination [24, 160]. It has been suggested that the transport is mediated by Mg ATP-dependent carrier or ATP-binding cassette (ABC) transporter [15].

The precipitous induction of PCs occurs inside cells as result of the varying levels of multiple types of HMs where PCs via sulfhydryl and carboxyl groups can attach to some HM cations and anions such as Cd, Cu, Ag, Zn, Pb, Ni, and Ar [155, 161, 162]. However, Cd^2+^ ions are found to be the most effective stimulator of PCs synthesis where they are 4- to 6-fold stronger in inducing PCs than Cu^2+^ and Zn^2+^ in cell cultures of* Rauvolfia serpentina* [163] and red spruce (*Picea rubens* Sarg), respectively [164]. PCs can be both produced and accumulated in roots and aerial organs. Nevertheless, the majority of studies suggest that they tend to be first biosynthesized and amass in roots. It has been shown that, in sunflower exposed to Cd intoxication, phytochelatins levels in roots were at least two times as much as those in leaves [165].

Fidalgo et al. [166] in* Solanum nigrum* L. showed that the production of PCs was enhanced in roots when the plant was exposed to 200 *μ*mol·L^−1^ Cu, which resulted in the immobilization of Cu excess in the root and its preclusion from moving toward the shoot. Batista et al. [167] concluded that the stimulation of different As-PC complexes in roots of some rice cultivars subjected to the elevated levels of arsenic reduced the transport of As from soil or root to the aerial parts and grains. These strategies can be effective in terms of preventing toxic metals from reaching the consumable parts of crops. On the other hand, some investigations show that when time variable is factored into the experiment and plants are exposed to the protracted HM stress, PCs-related activities as well as their concentration are increased in leaves. Heiss et al. [168] demonstrated that prolonged exposure of* Brassica juncea* to Cd resulted in 3-fold higher accumulation of PCs in leaves than roots. Szalai et al. [169] observed that treating maize plants with Cd for a longer period of time led to decreased PCs action in roots and increased level of phytochelatin synthase in leaves. They suggested that feedback regulation process or substrate reduction may be accountable for this phenomenon. In addition to the aforementioned factors, it seems that the type of plants in terms of their degree of tolerance to HM excess plays a role in determining PCs production, accumulation, and transportation site as well as their preferred movement path in plants. Zhang et al. [170] suggested that principal Cd detoxification mechanism in hyperaccumulator* Sedum alfredii* mediated by PCs occurs in shoots, which is similar to nonresistant plants. However, this event for wheat plant, as an efficient Cd accumulator, happened in roots [171]. In hyperaccumulators, it appears that they adopt mechanisms that involve long-distance translocation of PCs from root to shoot [172].

PCs chain lengths show variation within and among plant species as well as with HM types. Brunetti et al. [173] reported that PC_4_ was most pervasive oligomer in tobacco seedlings (*Nicotiana tabacum* L.) whereas PC_3_ was of higher concentration in* Arabidopsis*. In legumes, it is reported that PCs with longer chains are stronger binder to Pb in comparison to shorter PCs [174]. But there is no conclusive study to show whether the number of chains can have any impacts on the effectiveness of the PCs. Phytochelatins along with antioxidative enzymes can form a synergistic defensive regime in plants under HM stress which, in turn, can strengthen plant's resistance against metal intoxication. Chen et al. [152] demonstrated that the increased enzymatic biosynthesis of PCs coupled with the heightened activity of antioxidative system in* Brassica chinensis* L. led to an effective detoxification of Cd. A considerable effort has been made to identify and clone PCS genes that are responsible for the production of PCs.* Arabidopsis thaliana* phytochelatin synthase (*AtPCS1*) and wheat (*Triticum aestivum* L.) phytochelatin synthase (*TaPCS1*) were amongst the first plant PCS genes that were extracted [162]. The ongoing investigation into this area has led to the identification of various PCS genes in distinct plant species such as* Brassica juncea* (*BjPCS1*) and rice (*Oryza sativa* L.) (*OsPCS1*) [168, 175].

Real or synthetic expression of these genes in PC-deficient and transgenic plants or hyperaccumulators offer a very promising future for the possibility of increasing plants resistance against HMs and also phytoremediation strategies. In transgenic tobacco plants, artificial synthesis of phytochelatin gene enhanced their resistance to varying levels of cadmium [176]. Shukla et al. [154] showed transgenic* Arabidopsis* plants were much better HM accumulators than wild type* Arabidopsis* as a result of expressing synthetic phytochelatins (ECs). Guo et al. [177] demonstrated that overexpression of arsenic-phytochelatin synthase 1 (*AsPCS1*) and yeast cadmium factor 1 (*YCF1*) (isolated from garlic and baking yeast) in* Arabidopsis thaliana* resulted in an increased tolerance to Cd and As and also enhanced its ability to accumulate the metals to a greater extent.

### 3.2. Metallothioneins (MTs)

MTs, which were first extracted from equine kidney in 1957 [178], are another family of small cysteine-rich, low-molecular-weight cytoplasmic metal-binding proteins or polypeptides that are found in a wide variety of eukaryotic organisms including fungi, invertebrates, mammals, and plants as well as some prokaryotes [179–181]. Contrary to PCs that are the product of enzymatically synthesized peptides, MTs are synthesized as a result of mRNA translation [182]. Whereas PCs in plants may mainly deal with Cd detoxification, MTs appear to be capable of showing affinity with a greater range of metals such as Cu, Zn, Cd, and As [183]. MTs exhibit different characteristics and functionality based on their occurrence in different organisms; however, as our understanding towards the roles of plant MTs increases and given the fact that plant MTs are exceedingly varied in terms of their molecular properties and structural features [184], they are likely to have more and diverse functions in plant than any other living organisms. In plants, these ligands are involved in nullifying toxicity of HMs through cellular sequestration, homeostasis of intracellular metal ions, and metal transport adjustment [21, 185, 186]. In addition to their role in HM detoxification, MTs are known to be active agents in a number of cellular-related events including ROS scavenger [142], maintenance of the redox level [180], repair of plasma membrane [187], cell proliferation, and its growth and repair of damaged DNA [188]. There are a myriad of different endogenous and exogenous factors other than HMs that are able to induce the production and expression of MTs. Of these, osmotic stress, drought, extreme temperatures, nutrient deficiency, release of various hormones, natural and dark-induced tissue senescence, injuries, and viral infections can be mentioned [24, 179, 183].

Plants have multiple MT types that are generally divided into four distinct subgroups according to the arrangement of Cys residues [189]. They demonstrate patterns of organ and developmental stage specificity so that type 1 MTs are mainly expressed in roots, while the expression of type 2 MTs mostly occurs in shoots, type 3 MTs are induced in leaves and during fruit ripening, and type 4 MTs are abundant in the developing seeds [183, 186]. Regarding high level of sequence diversity of plant MT [190], each MT subgroup (MT1 to MT4) is further subdivided and referred to as isoforms. Guo et al. [185] subdivided sugarcane MT2 into three subclasses and termed them as MT2-1, MT2-2, and MT2-3 or in* Arabidopsis* MT4 are subdivided into MT4a and MT4b [21]. It seems that all four types of MTs and their isoforms identified in plants are able to bind to HMs and act as metal chelators or storehouse; however, mounting evidence suggests that on the one hand plant MTs show distinct treatment towards varying types of metals and on the other hand functionality of these plant MTs and their metal-binding and metal-affinity characteristics as well as tissue localization might be different within a plant species or among species.

Grennan [188] reported that, in* Arabidopsis*, there is every likelihood that MT isoforms from types 1 and 2 (1a, 2a, and 2b) and 3 are involved in copper chelation, while MTs isoforms from type 4 (4a and 4b) act as a zinc binder. García-Hernández et al. [191] showed that, in some mutants of* Arabidopsis*, MT1 may play a more important role in detoxifying copper in leaf veins than in leaf mesophyll. Yang et al. [192] showed that the induction of* OsMT1a* (*Oryza sativa* L. metallothionein type 1) was crucial to the zinc homeostasis in roots of rice. In grain-filling and mature seeds of barely, it was demonstrated that the primary function of MT3 is to maintain homeostasis of Zn and Cu, whereas MT4 was involved in storage of Zn [193]. In soybean, it was shown that MT1, MT2, and MT3 were more likely to get involved in detoxification of deleterious amounts of Cd, whilst MT4 exhibited Zn-binding characteristics [194]. It can be suggested that varying types of MTs and their isoforms have distinct and overlapping functions in homeostasis and HM detoxification [179]. More work still needs to be done to find out the possible reasons for these differential and preferential behavior of plant MTs towards metals; nevertheless, it appears that differences in genetic structure of plants, complex diversity in the metal binding regions of plant MTs [188], and different sequence and performance of isoforms [195] might be able to provide some explanation to the observed patterns. Overexpression experiments are very popular with plant MTs and expressing as well as engineering them through DNA recombinant methods into plants, yeasts, and bacteria that lack some of these proteins can increase our knowledge of MTs and their performance to a greater extent and also provide unique opportunities for phytoremediation or bioremediation strategies. Some works are suggestive of MTs promoting the capability of transgenic plants in terms of decreasing the production of reactive oxygen species and fortifying cellular antioxidant defense system when it comes to detoxifying excessive levels of HMs. Xia et al. [196] showed that expression of* Elsholtzia haichowensis* metallothionein type 1 (*EhMT1*) in tobacco plants not only increased the tolerance of transgenic tobacco to copper toxicity but also decreased the synthesis of hydrogen peroxide and improved peroxidase activity (POD) in roots, leading to enhanced ability of plants to cope with oxidative stress. Zhou et al. [9] demonstrated that although* TaMT3*, a metallothionein type 3 from* Tamarix androssowii*, engineered into tobacco resulted in increased tolerance to Cd stress through significant increases of SOD functionality, which raised the ability of ROS cleaning-up in transgenic plant, it led to decreased POD activity. It seems that the impact of the expressed metallothionein on distinct components of antioxidant system of transgenic plants is different, which requires further investigation. Ectopically expressed MTs in transgenic plants are shown to enhance their tolerance towards metal intoxication. Kumar et al. [197] showed that* OSMT1e-p*, a type 1 MT obtained from a salt tolerant rice genotype (*Oryza sativa* L. cv. Pokkali), imparted tolerance towards copper and zinc toxicity when ectopically expressed in transgenic tobacco. They observed that tobacco plants that had received the gene tended to retain excessive amounts of Cu^2+^ and Zn^+2^ in their roots or lower leaves, significantly reducing the HMs ions movement and content to/in upper foliage and harvestable organs. Zhigang et al. [198] concluded that the ectopic expression of* BjMT2*, a metallothionein type 2 from* Brassica juncea*, in* Arabidopsis thaliana* increased copper and cadmium tolerance at the seedling stage but acutely reduced root development when there was no heavy metal exposure. These trends may suggest that ectopic expression of MTs in transgenic plants may act in host plant in a nonspecific way and differently impact the organ growth.

### 3.3. Proline (Pro)

Pro is a proteinogenic five-carbon *α*-amino acid that acts as a compatible and metabolic osmolyte, a constituent of cell wall, free radical scavenger, antioxidant, and macromolecules stabilizer [94, 199, 200]. Some other functions of Pro include promoting embryo/seed evolvement, extending stem length as well as moving plants from vegetative growth to reproductive stage [201]. The production of elevated levels of Pro by higher plants is a typical nonenzymatic response to tensions caused by a wide range of biotic and abiotic stressors such as excessive salinity, drought, increased solar ultraviolet (UV) radiation, HMs, and oxidative stress [202]. In fact, Pro plays multifarious roles including adaptation, recovery, and signaling when it comes to combating stress in plant [166]. A number of mechanisms by which Pro increases the resistance of plants to HM toxicity have been proposed.

Clemens [203] suggested that HM-induced Pro accumulation in plants is not directly emanated from HM stress, but water balance disorder, which occurs as a result of metal excess, is responsible for the induction of Pro. In this regard, Pro functions as an osmoregulator or osmoprotectant. Mourato et al. [147] and Tripathi and Gaur [204] proposed that ROS scavenging by Pro, which is stimulated by HM stress, is primarily conducted through detoxifying hydroxyl radicals and quenching singlet oxygen. Increase in antioxidant enzyme activities, their protection, maintenance of cellular redox homeostasis [147], and reconstruction of Chl as well as regulation of intracellular pH [149] are associated with the activity of Pro when plants are exposed to HMs. It is reported that Pro can act as a metal chelator and protein stabilizer [187]. However, Tripathi and Gaur [204] in* Scenedesmus* sp. surveying the relationship between zinc and copper-induced stress and Pro accumulation did not support the notion that Pro functions as a metal chelator.

Literature review shows that the induction of Pro in plants in response to HM is to a great extent concentration-dependent, organ and metal specific. In hyperaccumulator* Cynara scolymus* L. (artichoke), it is demonstrated that there is a linear association between Pro augmentation in cells and HM concentration [205]. Gohari et al. [206] showed that Pro concentration in root of rape seed (*Brassica napus* L.) increased when the plant was exposed to rising concentrations of Pb^2+^ (100 to 400 *μ*M) but Pro accumulation at aerial parts was not as conspicuous as root. The same organ-specific accumulation of proline where roots contained more proline than shoots is exhibited in a plethora of experiments with different plants including* Brassica juncea* L. subjected to Pb and Cd stress [23],* Solanum nigrum* L. exposed to Cu stress [166], wheat subjected to Cd [207], and lemongrass (*Cymbopogon flexuosus* Stapf) subjected to Hg and Cd stress [208]. In hybrid poplar (*Populus trichocarpa* ×* deltoides*), it was shown that Pro accumulation in roots was almost 2-fold higher than that of leaves when Cd was applied in strong doses, but there was no significant difference between leave and root Pro content at lower concentrations of the metal [209]. However, contrary to the above-mentioned works, some reports indicate that Pro tends to more accumulate in shoots of HM-stressed plants than in roots [146, 210]. It seems that, in addition to type of plant species and preferential accumulation of metals within plant parts, condition and variables of experiments, such as heavy metal concentration, temperature, and duration of exposure as well as substances supplemented to experimental medium are crucial factors determining the way Pro augmented in plant parts. However, the rapid induction of Pro in roots and forming Pro-metal complexes may offer a better and effective way of nullifying toxicity of metals rather than allowing them to reach above-ground parts. Theriappan et al. [211] experimenting with cauliflower seedlings (*Brassica oleracea* var. botrytis) and three HMs (Cd, Hg, and Zn) noticed the concentration-dependent accumulation of Pro in which intensification of toxicity (up to 1000 *μ*M) almost doubled the production of Pro. Additionally, they showed that Hg was the strongest initiator of Pro. In another study using sal seedling (*Shorea robusta*), it was determined that Cd, Pb, and As were, respectively, stronger evokers of proline [212]. Kumar et al. [213] in wheat seedlings found that Cu was stronger inducer of Pro than Zn. Rastgoo et al. [149] studying effects of equimolar amounts (50 *μ*M and 100 *μ*M) of HMs (Cd, Co, Pb, and Ag) on Gouan (*Aeluropus littoralis*) found that maximum Pro accumulation occurred when the plant was treated with Cd. Zengin and Kirbag [210] showed that Pro content in sunflower seedlings subjected to various amounts of HMs was strongly induced in the order of Hg > Cd > Cu > Pb. The results obtained from these studies indicate that the capability of a specific trace metal to induce proline accumulation may depend on the concentration and specificity of HMs, their toxicity threshold, and plant species employed in the trials. As indicated by Ruscitti et al. [214] increasing HM concentration raises the content of cell Pro to a specific level, after which suppression of Pro accumulation occurs as the amount of metal increases beyond a certain threshold.

Spraying Pro on the foliar parts of plants grown under HM stress is shown to be an effective method to reduce the poisonousness of metals and give rise to the activation of protective mechanisms in plants in order to negate toxic effects of HMs. Hayat et al. [215] showed that exogenously applied Pro on cadmium stressed-chickpea enhanced the activity of antioxidative enzymes, carbonic anhydrase and photosynthetic parameters, which all contributed to the increased tolerance of the plant to Cd. Moreover, they observed that there was a sharp rise in the content of endogenously produced Pro when the plant received Pro from exogenous sources, thereby aiding it to better cope with the metal stress. Shahid et al. [216] showed that the exogenous application of Pro (pure synthetic proline or proline fortified with essential nutrients) on pea protected the plant against phytotoxic impacts of nickel by reducing lipid peroxidation and electrolyte leakage, heightening activities of polyamine biosynthetic enzymes and improving leaf polyamines and increasing concentration of endogenous compatible solutes. It was also concluded that Pro enriched with nutrients was more effective than pure Pro in enhancing plant growth under stress. Hayat et al. [217] reported that exogenous Pro can form complex with various metals such as Cu, Cd, and Zn in which it can overcome inhibition of nitrate reductase caused by metal toxicity. It is reported that Pro pretreatment can ameliorate the phytotoxicity of Hg^+2^ in rice by reducing ROS concentration [218]. Role of exogenous Pro in HM detoxification especially testing different enriched extracts containing Pro needs to be paid more attention. Furthermore, priming seeds with Pro for the purpose of increasing the tolerance of plants to HM toxicity has the merit of investigation.

### 3.4. Arbuscular Mycorrhizal (AM)

Symbiotic mycorrhizal fungi such as AM form a mutualistic symbiosis with roots of most vascular plant species under different climatic conditions in which they are beneficiary of photosynthetic assimilations provided by plants and in return they improve the mineral nutrition status of plants and can also enhance their tolerance towards some stresses and pollutants [219–221]. Plant-fungal mutualism may act as a precursor in which it signals the herald of stress to symbiotic plants so that they can make their protective mechanisms active to ameliorate deleterious effects of stress earlier than nonsymbiotic plants [214]. Although most of the discussions involving mycorrhizal symbiosis with plant roots in relation to HMs fall under the category of bioremediation methods, its multifarious and crucial services to plants make it inevitable to view this relationship from the protective aspects, which contributes to overall defensive systems of plants, in particular, against external stressors, like HMs. Principal mechanisms adopted by mycorrhizal fungi to cancel out impacts of HM stress on plants include acting as a barrier by depositing metals within cortical cells [222], binding metals to cell wall or mycelium as well as sequestering them in their vacuole or other organelles [12] releasing heat-shock protein and glutathione [223], precipitating or chelating metals in the soil matrix via producing glycoprotein or making phosphate-metal complexes inside the hyphae [224–226], and reducing the strength of metals by heightened root and shoot growth [227]. The varied strategies employed by AM when facing toxicity of HMs suggest that different species of mycorrhizal fungi might act specifically or adopt the remedial function which suits the prevailing condition in either rhizosphere or plant.

It was shown that in ryegrass (*Lolium perenne* L.), which has a symbiotic relationship with AM, the translocation of Cd, Ni, and Zn from soil to different parts of the plant was significantly reduced as a result of immobilization of HMs in soil [228]. The same result was obtained by Shivakumar et al. [229] when working with green gram (*Vigna radiata*) grown in soil containing excessive Zn. It is reported that the changes in pH soil due to the activities of AM fungi are a major contributing factor to the immobilization of metals in mycorrhizosphere region [230]. Huang et al. [231] showed that AM fungus (*Glomus mosseae*) decreased availability of excessive Zn, Cu, and Pb for maize growing in HM contaminated soil through binding the metals to organic matters or absorbing them into its organs, thereby limiting the possibility of metal uptake by host plant. When antioxidant defense machinery of plants exposed to elevated levels of HMs is exhausted as a result of induction of reactive oxygen species, AM association can reduce or prevent the induction of ROS species and also give a boost to the activity of detoxifying enzymes within plants. Abad and Khara [232] showed that wheat plants colonized by different AM fungi species subjected to toxic levels of Cd had conspicuously more functionality of protective antioxidants such as APX and GPX in their roots and shoots compared to non-AM wheat. They also observed that, among fungal species,* Glomus veruciforme* and* Glomus etunicatum* were the most effective activators of the protective proteins. The decrease in lipid peroxidation and electrolyte leakage and increased activities of superoxide dismutase (SOD), catalase (CAT), and peroxidase (POX) are observed in mycorrhizal pigeon pea (*Cajanus cajan* L.) plants under Cd and Pb-contaminated soils [233]. Farshian et al. [234] demonstrated that lettuce plants (*Lactuca sativa* L.) under ZnSO_4_ stress which were inoculated with AM fungus (*Glomus etunicatum*) had higher levels of cellular protein and, as a result, increased content of antioxidant enzymes compared to noninoculated ones, due to the fact that the AM fungus had sequestered Zn in its hyphae. However, chlorophyll and sugar content decreased in both AM and non-AM plants. In contrast, Rahmaty and Khara [235] observed that Cr-stressed maize plants treated with AM fungus (*Glomus intraradices*) had greater Chl content compared to maize plants that had not received AM treatment. It seems that metal or plant specificity is involved in this discrepancy of results. The information on the way by which AM fungi influence production and augmentation of other metabolites such as PCs, MTs, and Pro in heavy-metal stressed plants are rather scant and ambiguous. Abdelmoneim et al. [236] experimenting with Cu and Cd-stressed maize plants inoculated with two species of mycorrhizal fungi (*Glomus mosseae *and* Acaulospora laevis*) observed that there was a decline in Pro accumulation in AM infected maize compared to non-AM maize and the success of fungal and plant association in reducing deleterious effects of HM was attributed to other factors. However, Ruscitti et al. [214] showed that the interaction of mycorrhizal inoculation (*Glomus mosseae *and* Glomus intraradices*) and Cr-stressed pepper plants (*Capsicum annuum* L.) resulted in increased leaf Pro content but depressed root Pro concentration. Amanifar et al. [237] observed that shoot Pro concentration in mycorrhizal tomato (*Lycopersicon esculentum* L.) subjected to Pb treatment and inoculation of the above-mentioned* Glomus* species was not significantly affected when compared to control plants but there was a pronounced increase in root Pro content. They stated that observed different pattern of Pro induction in metal-stressed plants inoculated with AM may be due to fungal species, plant, and HM type. Moreover, it seems that growth condition, method of inoculation, and time of exposure to heavy metal intoxication may play a role in determining the way through which Pro is produced in the presence of AM. Further studies are needed to be done to determine the possible antagonistic or synergistic interaction between AM fungi and the protective metabolites which are copiously produced by metal-stressed plants. Major crops such as wheat, maize, and rice are exhibited to be the hosts of AM fungi [238], which necessitate the identification and propagation of proper fungus species that are efficient in increasing plant tolerance to HM toxicity.

## 4. Conclusion and Future Outlook

Contamination of soil and water by HMs in changing environment poses a serious threat to public and food safety and is now emerging as a major health hazard to humans and plants. This has become more accentuated and prominent as human-made disturbance of biological resources of the planet has accelerated the occurrence of many abiotic stresses for example, HMs. As a consequence, plants are now exposed to toxicity of HMs more than any time in their history since the beginning of their terrestrial life on planet earth. This necessitates making more efforts to deepen our appreciation of HMs and the way plants respond to their ever-growing presence. In current review, the various detrimental consequences of plant exposure to HM stress were discussed. This ranged from symptomatic and morphological manifestation of HM toxicity on the main organs of plants to inter- and intracellular intoxication.

This review showed that HMs, irrespective of their redox-associated mode of action, are capable of disrupting prooxidants/antioxidants equilibrium in plant cells, tilting the balance in favor of the latter, inducing ROS, and directly reacting with functioning cellular macromolecules and organelles. Moreover, replacement of the essential cations with the toxic HM ions and their attachment to active groups of cofactors are common degenerative phenomena caused by HMs stress. This explains why there is a remarkable resemblance between the visual symptoms which occur in HM-stressed plants and the ones suffering from dearth of essential nutrients. It is evident that, in addition to plant type variation and HM threshold limit concentration, edaphic and light conditions are of key factors determining the occurrence, intensity, and toxicity of HMs. Also, uptake, mobility, and translocation of HMs within plant tissues or cells are greatly dependent on plant species, HM type, and concentration as well as oxidation state of HMs. We described some diverse defense procedures employed by plants when encountering deleterious impacts of HMs. All plant species, either tolerant or sensitive to HM stress, possess a basic defense system which gets activated upon the perception of threat from HMs. Commendable advances and progress achieved in molecular and biological fields have shed some light on the understanding of some complex strategies used by plants at cellular and molecular levels to combat metal stress. In this regard, functional diversity and molecular versatility of PCs and MTs are becoming intriguing when it comes to HM detoxification and maintaining cellular ion balance. Their role seems to go beyond being mere HM-chelating peptides or HM vacuolar and cellular sequestrators. They may act as cellular homeostatic or detoxifying agents. In particular, PCs and MTs are likely to interact directly or indirectly with plant antioxidant defense system or get involved in translocating and distributing excessive ion metals between root and shoot in a time or tissue-specific manner. Moreover, there are obvious indications that use of transgenic plants overexpressing PCs or MTs confers substantial HM tolerance. Therefore, transgenic and candidate gene approaches can be effectively adopted for phytoremediation purposes or for the fortification of plants that are deficient in PCs or MTs. This review also demonstrated that multifunctionality of Pro in aiding plants to tolerate HM stress is considerable since it can exhibit both chelating and antioxidant-related activities; however, its functions and effectiveness are immensely varied based on HM type and concentration and also according to plant variety as well as organ and tissue types. We explained that the contribution of AM symbiosis to plant defense system against HM stress is indispensable so that it may encompass or regulate many HM defense activities such as HM stress signaling, chelating, ion homeostasis control, and compatible solutes augmentation. Furthermore, AM and some antioxidant components such as SOD, APX, and CAT are likely to act in an integrated manner at excessive levels of HMs and raise plant tolerance to HM stress. However, although possible interrelatedness between AM and Pro is reported, a definite collaboration of AM with Pro may not yet be elucidated.

Literature review indicates that there are some areas that are needed to be explored more thoroughly. Under the condition imposed by climate change, water from soil is depleted at faster rate than ever and intensity of some abiotic stresses is increased. Therefore, it is essential to examine the impacts of HM stress on plants by simultaneous application of several stress factors such as heat, drought, light, and salinity. In addition, the elevated levels of atmospheric trace gases and their possible links to HM stress should be investigated. This will also provide a comprehensive assessment of responses and evaluation of the effectiveness of transgenic plants to HMs under climate change circumstance. Since most of our information concerning plant defense mechanisms against heavy metal toxicity comes from adult plants, it is important to conduct more studies with young plants in order to compare and contrast between their defense system and adult plants against HM stress. The relationship between plant antioxidative defense mechanisms and HM chelators such as PCs or MTs ligands also needs to be well defined and established. This becomes more important as MTs exhibit antioxidant activities. There is no clear evidence how activities amongst these apparently separate defense systems are coordinated and whether they act synergistically or antagonistically in relation to antioxidative systems when plants are confronted with metal toxicity.

Exogenous application of various organic or inorganic compounds and their possible ameliorative effects on HM-induced toxicity in plants signify a promising future. In addition to Pro, it is shown that exogenously applied nitric acid (NO) and salicylic acid (SA) have protective effects against deleterious impacts of HMs [98, 239]. It is essential to improve our understanding of the exact mechanisms involved in the actions of such biological molecules and the level of their interaction with different plant species in alleviating adverse effects of HMs. There is a great need to find out how HMs affect crop plants in low input sustainable farming practices where there is a considerable emphasis in terms of supplying soil with organic fertilizers like compost with the objective of maintaining and boosting the association between naturally occurring or artificially introduced mycorrhiza and plants. Furthermore, examination and selection of suitable AM species for an efficient symbiotic relationship with plants towards combating HM stress are required to be done in an extensive manner.
